# Preparation of chitosan/MCM-41-PAA nanocomposites and the adsorption behaviour of Hg(II) ions

**DOI:** 10.1098/rsos.171927

**Published:** 2018-03-28

**Authors:** Yong Fu, Yue Huang, Jianshe Hu

**Affiliations:** Center for Molecular Science and Engineering, College of Sciences, Northeastern University, Shenyang 110819, People's Republic of China

**Keywords:** hybrid mesoporous composite material, chitosan, diatomite, Hg(II) ions, adsorption

## Abstract

A novel functional hybrid mesoporous composite material (CMP) based on chitosan and MCM-41-PAA was reported and its application as an excellent adsorbent for Hg(II) ions was also investigated. Innovatively, MCM-41-PAA was prepared by using diatomite and polyacrylic acid (PAA) with integrated polymer–silica hybrid frameworks, and then CMP was fabricated by introducing MCM-41-PAA to chitosan using glutaraldehyde as a cross-linking agent. The structure and morphology of CMP were characterized by X-ray diffraction, Fourier transform infrared spectra, thermogravimetric analysis, scanning electron microscopy and Brunauer–Emmett–Teller measurements. The results showed that the CMP possessed multifunctional groups such as –OH, –COOH and –NH_2_ with large specific surface area. Adsorption behaviour of Hg(II) ions onto CMP was fitted better by the pseudo-second-order kinetic model and the Langmuir model when the initial Hg(II) concentration, pH, adsorption temperature and time were 200 mg l^−1^, 4, 298 K and 120 min, respectively, as the optimum conditions. The corresponding maximum adsorption capacity could reach 164 mg g^−1^. According to the thermodynamic parameters determined such as free energy, enthalpy and entropy, the adsorption process of Hg(II) ions was spontaneous endothermic adsorption.

## 1. Introduction

Hg(II) ion is regarded as one of the most toxic heavy-metal ions [1]. The wastewater from many industries involved in mercury lamps, paint and chloralkali production is a source of Hg(II) ion pollution with its release to the ecosystem [2,3]. Owing to its highly soluble properties, emission of Hg(II) ion not only pollutes the water source and the soil, but also Hg(II) ion is easily converted to organic Hg(II) and methylmercury, becomes enriched in concentration in the body of microorganisms and is finally accumulated in the human body through the food chain [4–6]. With its bioaccumulation on entering the bloodstream and spreading throughout the body, it is toxic to brain, liver, kidney, bones and lungs of humans, resulting in symptoms of mercury poisoning, such as hair loss, limb numbness, insomnia and more dreams, central nervous system disorders, and vision and hearing loss [3,7,8]. The maximum permissible concentration of Hg(II) stipulated in drinking water by the World Health Organization is 6 µg l^−1^, while the corresponding level recommended by the US Environmental Protection Agency is reduced to 2 µg l^−1^ [9,10]. In view of the hazards of the Hg(II) ion and the maximum permissible limit, looking for efficient approaches to remove Hg(II) ions has been a challenging task. Compared to other methods applied for the uptake of mercury ions such as chemical precipitation [11], electrolysis [12], ion-exchange [13] etc. up to now, the adsorption method is the most popular treatment for the removal of Hg(II) ions because of significant merits such as high efficiency, simplicity and economy [14–16].

As known, the adsorption capacity and removal rate are closely related to the performance of the adsorbent, so a large number of materials such as activated carbon [17], carbon nanotubes [18], natural or modified diatomite [19], chitosan (CS) [20,21] and synthetic mesoporous silica [22] have been explored widely for the removal of various pollutants.

CS and its derivatives as natural polymers have attracted considerable attention because of renewability, easy availability and non-toxicity [23]. CS has outstanding removal capacity with regard to many mercury ions especially the Hg(II) ion, which is attributed to amine and hydroxyl groups with chelating metal properties. However, CS can be dissolved in acidic solutions, resulting in agglomeration and formation of a gel, which makes CS difficult to disperse and hinders many hydroxyl and amino groups from chelating metal ions [24]. To improve the acid resistance and further increase the adsorption capacity, natural CS is modified by some physical or chemical approaches [25,26].

MCM-41, as one of the most famous ordered mesoporous silica materials since the discovery of M41S silica in 1992 [27], has been commonly applied for the adsorption of heavy-metal ions [28,29], owing to large surface areas, regular pore structure and adjustable pore sizes [30,31]. Up to now, noticeably, tetraethyl orthosilicate (TEOS), as a silica source for preparation of most mesoporous materials is usually expensive and toxic to the environment, which is inconsistent with the requirements of green chemistry [32]. Compared with TEOS, diatomite is cheap and rich in nature because it is a siliceous sedimentary rock largely consisting of amorphous silica [33]. Sanhueza *et al*. [34] synthesized ZSM-5 using diatomite as a silica source under hydrothermal conditions. In addition, MCM-41 with a pure silicon skeleton has some disadvantages such as unstable structure and poor adsorption capacity; in order to improve the physical and chemical properties of the material, the structure and surface of MCM-41 are modified to meet specific practical requirements [35]. In this aspect, constructing organic–inorganic hybrid frameworks to enhance the properties of MCM-41 has attracted more and more interest [36,37]. For instance, Mohammadnezhad *et al*. [38] fabricated MCM-41/PS for adsorption of Cd(II). Li *et al.* combined the advantages of MCM-41 and molecularly imprinted polymers to improve the adsorption capacity of mesoporous materials [39].

It is, therefore, crucial to consider the compatibility of mesoporous materials and polymers in order to enhance the properties of nanocomposites. With regard to the blending of a polymer containing a desired functional group to chelate metal ions and a silica precursor, polyacrylic acid (PAA) is a good candidate because of its amphiphilic character, mesostructural ordering capability with cationic surfactants and carboxylic acid functional groups to chelate heavy-metal ions [36].

**Figure 1.**
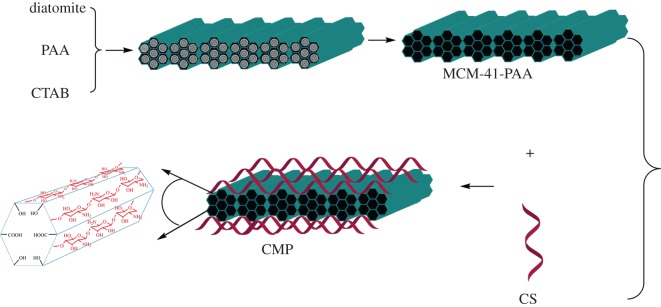
Schematic depiction of the formation of **CMP**.

To the best of our knowledge, no research on CS/MCM-41-PAA nanocomposites has been reported. The purpose of the present work, considering the interesting properties of CS, MCM-41 and PAA, firstly, was to fabricate MCM-41-PAA using diatomite as a silica source under hydrothermal conditions. Secondly, MCM-41-PAA was introduced to a CS gel solution and cross-linked with glutaraldehyde (GA). Finally, new CS/MCM-41-PAA nanocomposites (**CMP**) were successfully prepared. The optimal sorption conditions, adsorption kinetics, thermodynamic properties and reusability were explored by investigating the effect of different parameters such as adsorbent dosage, initial pH, initial metal ion concentration, contact time, temperature and number of recycles. In addition, this work also demonstrated some new insights into using cheap diatomite as the silica source to obtain mesoporous materials, and that constructing organic–inorganic hybrid frameworks with polymer materials further improved the properties of mesoporous materials. The schematic depiction of the formation of **CMP** is illustrated in figure 1.

## 2. Material and methods

### 2.1. Materials

CS (85% de-acetylated) was purchased from Qingdao Baicheng Biochemical Co. (Qingdao, China). The raw diatomite (92.8% SiO_2_, 4.2% Al_2_O_3_, 1.5% Fe_2_O_3_ and other metal oxides) was purchased from Jilin Kaida Diatomite Co. Ltd (Jilin, China). Cetyltrimethylammonium bromide (CTAB) and GA (25%) were purchased from Damao Chemical Agent Company (Tianjin, China). PAA (30%, *M*_w_ = 3000) was purchased from Tianjin Kemiou Chemical Reagent Co., Ltd. Mercurium nitrate was purchased from Guizhou Tongren Tailuier chemical plant (Guizhou, China).

### 2.2. Characterization

Fourier transform infrared (FT-IR) spectra were obtained using a PerkinElmer Spectrum One (B) spectrometer (PerkinElmer, Foster City, CA) with the wavelength range between 500 cm^−1^ and 4000 cm^−1^ using a KBr pellet. The morphology of samples was examined by electron scanning microscopy (SEM, JEOL 6500F, Japan). The X-ray diffraction (XRD) data were obtained by an XRD powder diffractometer (D8 Advance, Bruker, Germany) using Cu-K*_α_* radiation (*λ *= 1.54 Å) between 1° and 10° (2*θ*) at 40 kV, 40 mA. Thermogravimetric analysis (TG) was performed with a TG instrument (Netzsch 209C, Hanau, Germany) at a heating rate of 20°C min^−1^ from 40 to 600°C under N_2_ flow. The Brunauer–Emmett–Teller (BET) and Barret–Joyner–Halenda (BJH) methods were used to determine the surface area and pore size distribution of nanocomposites in N_2_ adsorption–desorption (Quadrasorbsi, Quantachrome, USA) experiments at 77 K.

### 2.3. Preparation of MCM-41-PAA

Based on diatomite, CTAB and PAA as a silica source, a template and the MCM-41 hybrid framework, MCM-41-PAA was prepared according to the following procedure. Firstly, diatomite (2.70 g) was added to a solution of NaOH (1.02 g, 25 ml of distilled water). After heating at 150°C for 5 h, the precursor was transferred to solution of CTAB (3.06 g, 51 ml of distilled water) under stirring. A 2.7 g aliquot of a 30% PAA solution was added drop-wise to the mixed solution, and then several drops of ethanol were added drop-wise and vigorously stirred for 1 h. The pH of the mixture was adjusted to 10 with 2 mol l^−1^ H_2_SO_4_ and diverted into a stainless steel autoclave at 100°C for 24 h. Then, the precipitate of the autoclave was centrifuged and dried. The product (1 g) was moved to a solution of NH_4_NO_4_ (0.3 g) in ethanol (50 ml) as an extracting agent to remove the template; the mixture was heated to 78°C for reflux and extraction of 12 h, and centrifuged and dried. Finally, MCM-41-PAA was obtained with repetition of the above-mentioned operation two times.

### 2.4. Preparation of CS/MCM-41-PAA nanocomposites

The MCM-41-PAA (2 g) was dissolved in 1% acetic solution (50 ml), with formation of homogeneous suspension by stirring. CS powder (0.5 g) was added to the mixture and heated at 45°C under vigorous stirring for 30 min to increase the homogeneity, and then cross-linked with adding 1% of GA solution (0.39 ml) drop-wise under constant stirring for 4 h. The pH of the mixture was adjusted to 7 with 2 mol l^−1^ NaOH and then distilled water; then the precipitate was dried and ground into powder.

### 2.5. Adsorption experiments

To investigate the adsorption capacity and removal rate of **CMP** for adsorption of Hg(II) ions, stock solutions of standardized Hg(II) ions (1000 mg l^−1^) were configured from Hg(NO_3_)_2_; other initial concentrations of Hg(II) ions were obtained by further dilution. The pH of the solution was adjusted from 1 to 6 with 0.8 mol l^−1^ HNO_3_. The adsorption experiments were performed as follows: **CMP** (0.1 g) was added to a 200 mg l^−1^ Hg(II) ion solution (100 ml) in a 250 ml beaker placed on a magnetic stirrer at 600 r.p.m. for 3 h to ensure equilibrium. The concentration of Hg(II) ions after adsorption was calculated by using dithizone spectrophotometry. Under acidic conditions, Hg(II) ions formed an orange complex with dithizone, and the absorption of the complex was determined at *λ*_max_ = 485 nm, so the adsorption capacity and removal rate of **CMP** were obtained by the following equations:

2.1 $$q_{e}=\frac{(C_{0}-C_{e})\cdot V}{m}$$

and

2.2 $$\eta =\frac{\left(C_{0}-C_{e}\right)}{C_{0}}\cdot 100\%,$$

where *C*_0_, *C*_e,_ *V*, *m*, *q*_e_ and *η* are the initial and equilibrium concentrations of Hg(II) ion (mg l^−1^), volume of solution (l), sorbent dosage (g), adsorption capacity (mg l^−1^) and removal rate (%), respectively.

## 3. Results and discussion

### 3.1. Characterization of **CMP**

#### 3.1.1. Fourier transform infrared spectra analysis

**Figure 2.**
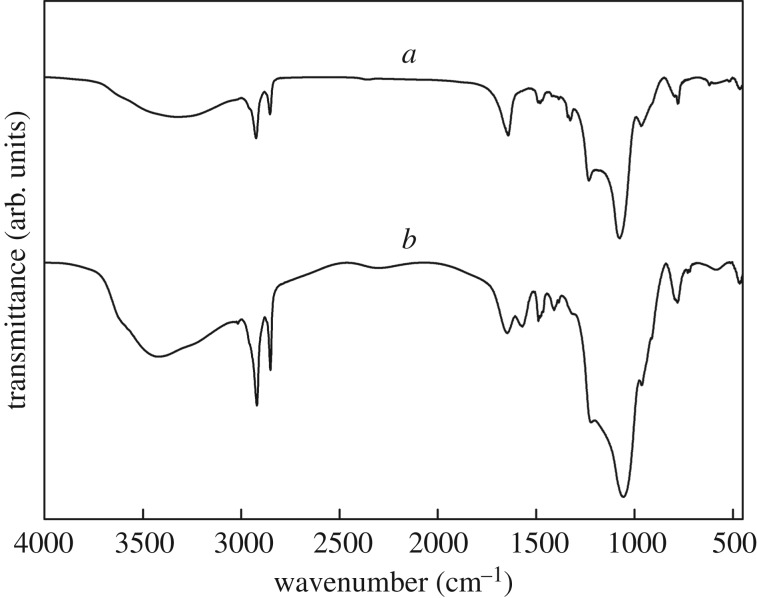
FT-IR spectra of MCM-41-PAA (*a*) and **CMP** (*b*).

The FT-IR spectra of MCM-41-PAA and **CMP** are shown in figure 2. Figure 2*a* represents the characteristic peaks of MCM-41-PAA. The peak at 3664 cm^−1^ was attributable to the stretching vibration of O–H (in –COOH group). In addition, the stretching vibration of *v*_as_ CH (in –CH_2_ group), *v*_sy_ CH (in –CH_2_ group) and C=O (in –COOH group) could also be observed at 2930, 2860 and 1650 cm^−1^, respectively. The peaks at 1470 and 1330 cm^−1^ were assigned to CH of the bending mode and C–O of the stretching mode, respectively, which are characteristic peaks of PAA. The peaks at 1090 and 806 cm^−1^ showed the stretching vibration of Si–O, which correspond to characteristic peaks of SiO_2_.

Figure 2*b* shows the FT-IR spectrum of **CMP**. Besides the SiO_2_ characteristic peaks and PAA, two new peaks at 1635 and 1598 cm^−1^ could be attributed to the C–O stretching vibration of NHCO and the N–H bending of NH_2_; the broad peak at 3429 cm^−1^ originated from overlapping vibration of O–H and N–H because the –NH_2_ absorption band shifted to a lower value.

#### 3.1.2. Scanning electron microscopy analysis

**Figure 3.**
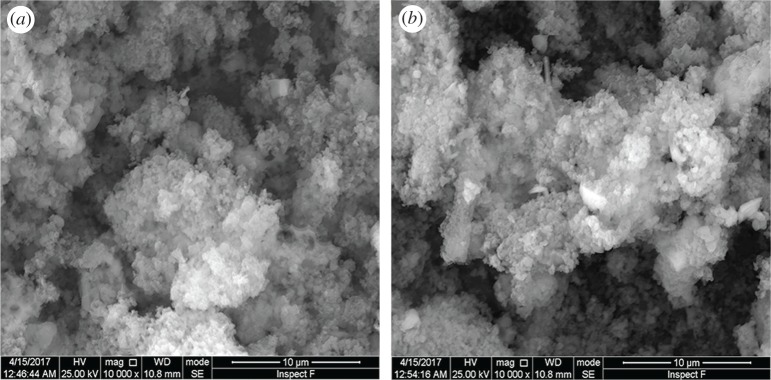
SEM images of MCM-41-PAA (*a*) and **CMP** (*b*).

The SEM images of MCM-41-PAA and **CMP** are shown in figure 3. Figure 3*a* demonstrates an interconnection of the nearly spherical particles with size in the range of 50–100 nm for MCM-41-PAA. As can be clearly seen in figure 3*b*, the white striped CS was homogeneously dispersed with the mesoporous material, forming an interesting monolithic structure.

#### 3.1.3. X-ray diffraction analysis

**Figure 4.**
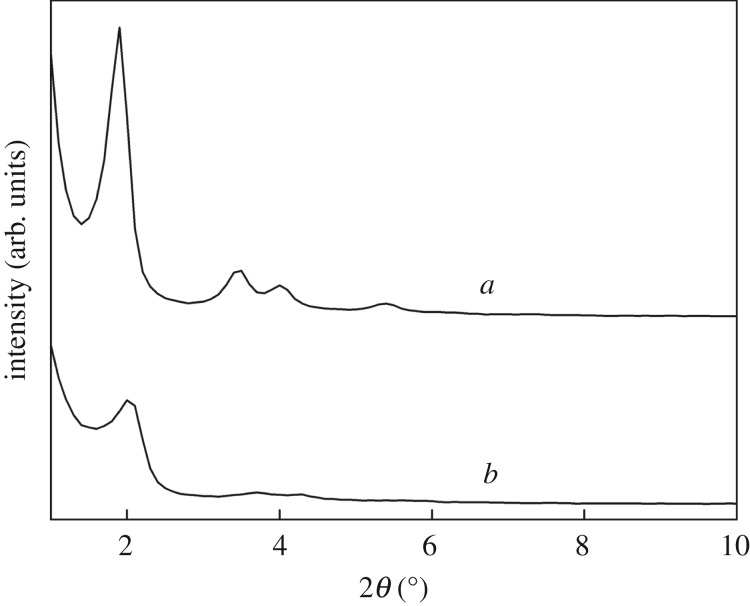
X-ray diffraction patterns of MCM-41-PAA (*a*) and **CMP** (*b*).

XRD patterns for MCM-41-PAA and **CMP** are shown in figure 4. The obtained characteristic diffraction peaks for MCM-41-PAA (figure 4*a*) are consistent with those in the literature; this indicated that the mesomorphic orders of MCM-41 remain intact on inclusion of PAA [40,41]. Compared with XRD diffractograms of MCM-41-PAA, however, the apparent decrease in intensity of the (100) diffraction peaks for **CMP** in figure 4*b* and other peaks were not observed, suggesting that the ordered structure of nanocomposites was slightly changed due to change of the inherent order caused by the mixing of MCM-41-PAA and CS.

#### 3.1.4. Thermogravimetric analysis

**Figure 5.**
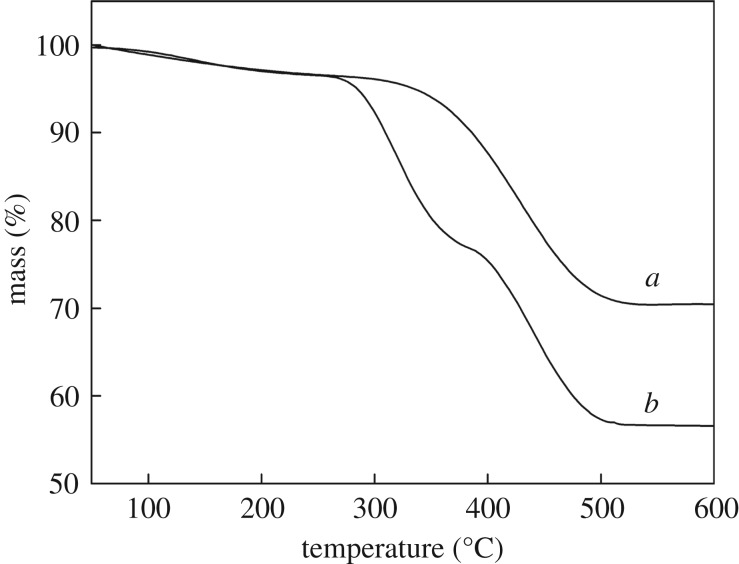
TG curves of MCM-41-PAA (*a*) and **CMP** (*b*).

Figure 5*a,b* shows TG curves of MCM-41-PAA and **CMP**. The first loss in weight at about 40–150°C was ascribed to physisorbed water. From the curve of MCM-41-PAA (figure 5*a*), the second loss in weight of about 28% between 150 and 600°C resulted from thermal decomposition of PAA. As shown in figure 5*b*, the loss in weight of 22–24% observed from the curve of **CMP** at the range of 150–450°C was attributed to the loss of CS and PAA; the remaining mass loss of PAA (18–20%) occurred at the range of 150–450°C. The final stage of all the curves did not show any significant loss in weight, which demonstrates MCM-41 possesses excellent thermal stability.

#### 3.1.5. Nitrogen adsorption–desorption isotherms

Textural characteristics of MCM-41-PAA and **CMP** including the total pore volumes (*V*_total_, cm^3^ g^−1^), the pore diameters (*D*_BJH_, nm) and the BET surface area (*S*_BET_, m^2^ g^−1^) were measured using N_2_ physisorption techniques at 77 K, and are summarized in table 1. Compared with MCM-41-PAA, the specific surface area reduced from 701.85 to 253.31 m^2^ g^−1^; the pore volume also reduced from 0.49 to 0.26 cm^3^ g^−1^, while the pore size increased from 3.59 to 3.71 nm. In addition, according to electronic supplementary material, figure S1 and figure S2, new holes were formed on the surface of MCM-41-PAA because CS was cross-linked with GA. These results demonstrated that the **CMP** still had a large specific surface area, and the pore size and numbers increased slightly after combining with CS.

**Table 1.** Surface area, pore diameter and pore volume of MCM-41-PAA and **CMP**.

sample	pore volume (cm^3^ g^−1^)	pore size (nm)	surface area (m^2^ g^−1^)
MCM-41-PAA	0.49	3.59	701.85
**CMP**	0.26	3.71	253.31

### 3.2. Effect of adsorption behaviour of Hg(II) ions on CMP

#### 3.2.1. Effect of pH value on adsorption

**Figure 6.**
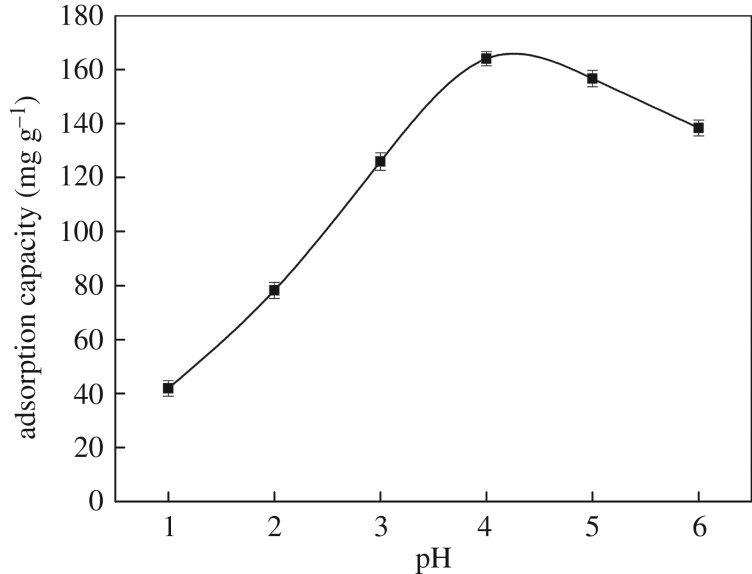
Effect of pH on the sorption of Hg(II) ions (200 mg l^−1^ initial Hg(II) ions concentration, 100 ml; **CMP**, 0.1 g; temperature, 298 K; time, 120 min).

Aqueous phase pH is the dominant parameter to optimize, because pH values affect the surface charge and metal-binding sites of an adsorbent and the degree of ionization [42]. To investigate the influence of pH on the removal of Hg(II) ions, all the experiments were performed under an initial pH range of 1–6, and the corresponding results are presented in figure 6. It could be clearly seen that the maximum adsorption was at pH = 4 for Hg(II) ions. There was a competition between Hg(II) ions and H^+^ ions on the surface of **CMP** at lower pH (pH < 4), which leads to the protonation of surface functional groups, with loss of the binding sites to chelate Hg(II) ions with an increase in acidity, resulting in a sharp decline in the adsorption capacity [43]. At higher pH (pH > 4), the hydroxide precipitation of Hg(II) ions might be formed on the surface and mesoporous channels of **CMP**, which will block the pores, decrease the retention and prevent further adsorption [35].

#### 3.2.2. The effect of contact time and temperature

**Figure 7.**
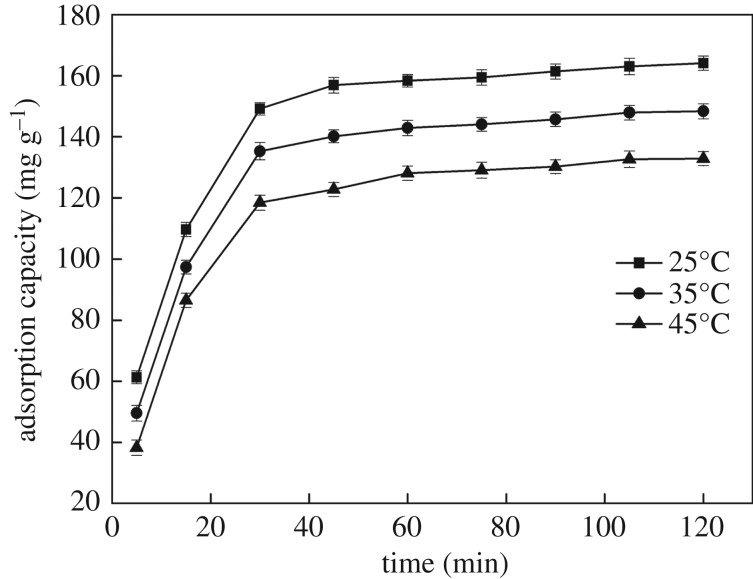
Effect of contact time and temperature on the sorption of Hg(II) ions (200 mg l^−1^ initial Hg(II) ion concentration, 100 ml; **CMP**, 0.1 g; pH, 4).

The influence of contact time on the adsorption capacity of Hg(II) ions was investigated, and the corresponding results are shown in figure 7. The adsorption capacity increased rapidly with contact time from 0 to 60 min because numerous surface active groups such as –NH_2_, –OH and –COOH of the adsorbents played a very important role in the adsorption of Hg(II) ions. Then the adsorption capacity continued to increase slowly by only relying on adsorption sites inside the pore structure, due to the saturation of the available adsorption sites, decrease of Hg(II) ion concentration and the presence of diffusion resistance, and finally reached saturation at 120 min.

### 3.3. Sorption kinetics

To define the efficiency of sorption, the rate of adsorption can be described by studying the kinetics of adsorption. According to the effect of contact time at the temperatures of 298, 308 and 318 K, the data of Hg(II) ions adsorbed are fitted by pseudo-first order [44] and pseudo-second order models [45], and both models may be expressed by the following equations:

3.1 ln ⁡(qe−qt)=ln ⁡qe−k1t

and

3.2 $$\frac{t}{q_{t}}=\frac{1}{k_{2}q_{e}^{2}}+\frac{1}{q_{t}}t,$$

where *q*_e_ (mg g^−1^) and *q*_*t*_ (mg g^−1^) are the adsorption amounts at equilibrium and at contact time *t*, and *k*_1_ and *k*_2_ are the first- and second-order kinetic rate constants, respectively.

**Figure 8.**
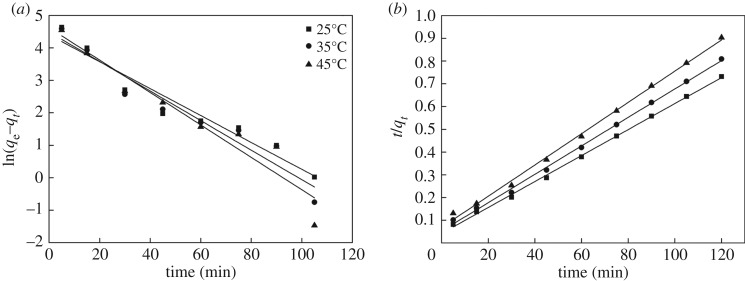
(*a*) Pseudo-first-order and (*b*) pseudo-second-order kinetic plots at different temperatures for the sorption of Hg(II) ions (200 mg l^−1^ initial Hg(II) ion concentration, 100 ml; **CMP**, 0.1 g; pH, 4; temperature, 298 K; time, 120 min).

The linear plots and the parameters of adsorption kinetics determined from the slope and intercepts are illustrated in figure 8 and table 2. It could be seen that the correlation coefficients (*R*^2^) at different temperatures of the second-order kinetic model were higher than 0.99, while the corresponding values of the first-order kinetic model were less than 0.95. In addition, the values of calculated *q*_e_ from the pseudo-second-order model were consistent with the experimental ones.

**Table 2.** Adsorption kinetic parameters of Hg(II) ion adsorption onto **CMP**.

		the pseudo-first-order model			the pseudo-second-order model		
temperature (K)	*q*_e−exp_^a^ (mg g^−1^)	*q*_e−cal_^b^ (mg g^−1^)	*k*_1_ (min^−1^)	*R*^2^	*q*_e−cal_^b^ (mg g^−1^)	*k*_2_ (g mg^−1^ min^−1^)	*R*^2^
298	164.06	81.49	0.0414	0.931	175.13	7.74 × 10^−3^	0.998
308	148.35	89.12	0.0455	0.923	160.00	7.46 × 10^−3^	0.998
318	131.64	101.49	0.0499	0.910	145.56	6.89 × 10^−3^	0.997

^a^Data obtained by experiment.

^b^Data obtained by calculation.

The obtained results indicate that the adsorption process of Hg(II) ions onto **CMP** follows the pseudo-second-order kinetic model, demonstrating that the adsorption of Hg(II) ions is chemical adsorption by valence forces via exchange or sharing of electrons between functional groups of **CMP** such as amine, carboxyl and hydroxyl and Hg(II) ions.

### 3.4. Sorption isotherm models

The interaction mechanism between adsorbate and adsorbent at equilibrium time is explored by Freundlich and Langmuir models [46,47]. In this work, the data of the equilibrium adsorption were determined by changing the initial Hg(II) ion concentration from 100 to 300 mg l^−1^, and were fitted by the Freundlich and the Langmuir models. The Freundlich and Langmuir isotherm equations could be represented by the following:

3.3 $$q_{e}=K_{F}{C_{e}}^{1/n}$$

and

3.4 $$q_{e}=\frac{q_{m}K_{L}C_{e}}{1+K_{L}C_{e}},$$

where *q*_e_ (mg g^−1^) represents the adsorption capacity at equilibrium, *q*_m_ (mg g^−1^) is the maximum adsorption capacity, *C*_e_ (mg l^−1^) is the Hg(II) ion concentration at equilibrium, *K*_F_ (mg g^−1^) and *K*_L_ (l mg^−1^) express the Freundlich and Langmuir constants, respectively, and *n* reflects the adsorption intensity.

**Figure 9.**
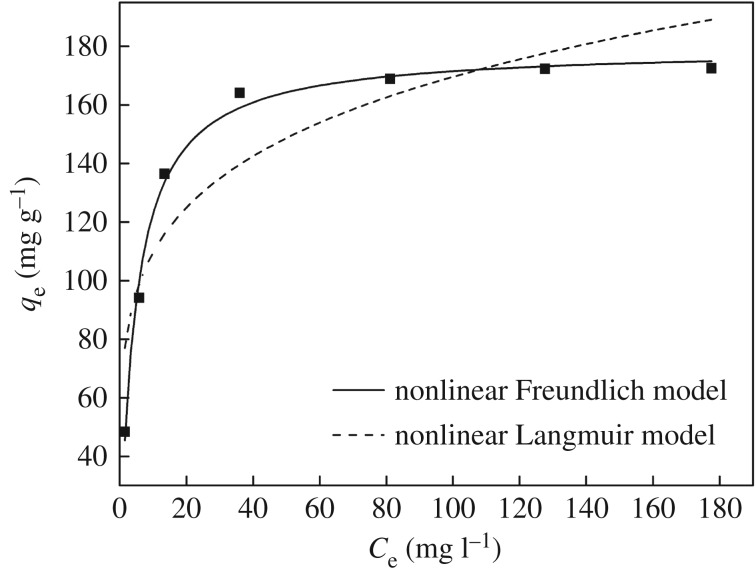
Nonlinear Langmuir and nonlinear Freundlich isotherm plots for the sorption of Hg(II) ions (**CMP**, 0.1 g; pH, 4; temperature, 298 K; time, 120 min).

Nonlinear regression analysis of the Langmuir and Freundlich isotherm models is illustrated in figure 9; the corresponding parameters of sorption isotherm models calculated are listed in table 3. In comparison with the Freundlich isotherm model, the Langmuir isotherm model was more suitable to describe the interaction mechanism of Hg(II) ion adsorption onto **CMP**, because the correlation coefficients (*R*^2^) were higher than 0.99 and *q*_m_ calculated by the Langmuir isotherm model was closer to the experimentally measured equilibrium adsorption capacity. It was thought that the adsorption of Hg(II) ions occurred at the identical limited number of monolayer adsorption sites on the surface of the adsorbent; firstly, Hg(II) ions could transfer from the solution to **CMP** by bulk diffusion and intraparticle diffusion, and then were adsorbed by chemical complexation at the active sites.

**Table 3.** Langmuir and Freundlich isotherm parameters.

	Langmuir			Freundlich	
*q*_m_(mg g^−1^)	*K*_L_	*R*^2^	*n*	*K*_F_	*R*^2^
179.43	0.2147	0.993	4.6436	70.71	0.811

### 3.5. Sorption thermodynamics

The effect of temperature on the adsorption of Hg(II) ions onto **CMP** was investigated at 298, 308 and 318 K; related thermodynamic parameters including enthalpy (Δ*H*^0^), entropy (Δ*S*^0^) and Gibbs energy (Δ*G*^0^) were obtained based on the following equations:

3.5 $$\ln\left(\frac{q_{e}}{C_{e}}\right)=\frac{-\Delta H^{0}}{RT}+\frac{\Delta S^{0}}{R}$$

and

3.6 $$\Delta {\text{G}}^{0}=-RT\cdot \ln\left(\frac{q_{e}}{C_{e}}\right),$$

where *R* (8.314 J mol^−1^ K^−1^) represents the ideal gas constant and *T* (K) is the absolute temperature.

**Figure 10.**
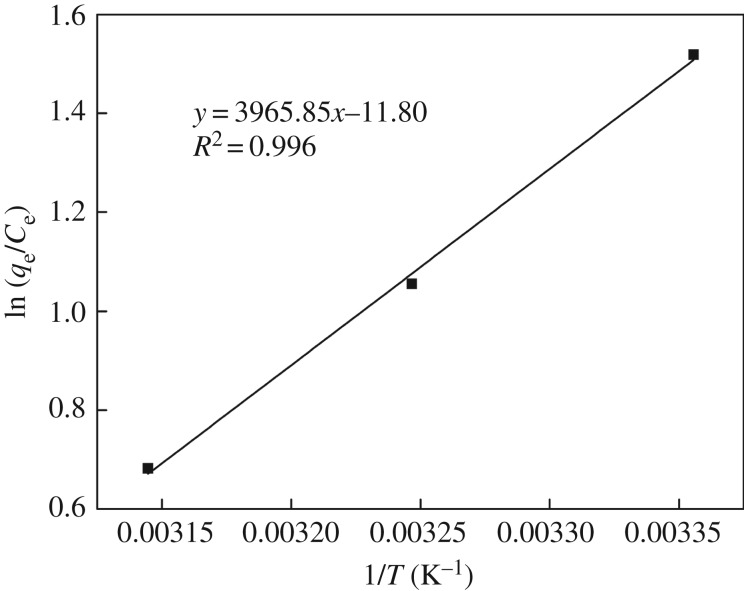
Plot of dependence of ln(*q*_e_/*C*_e_) on 1/*T* for the estimation of thermodynamic parameters (200 mg l^−1^ initial Hg(II) ions concentration, 100 ml; **CMP**, 0.1 g; pH, 4; time, 120 min).

The straight line obtained by plotting ln(*q*_e_/*C*_e_) versus 1/*T* is shown in figure 10. According to the slope and intercept of the line, Δ*H*^0^ (−32.97 kJ mol^−1^) and Δ*S*^0^ (−98.11 J mol^−1^ K^−1^) were determined; the negative value of Δ*H*^0^ suggests the exothermic nature of the whole adsorption process, which is consistent with the fact that the adsorption capacity of **CMP** decreases with increase in temperature. Based on equation (3.6), values of Δ*G* were estimated to be −3.76, −2.70 and −1.80 kJ mol^−1^ for 298, 308 and 318 K, respectively, and the numerical value of Δ*G*^0^ decreased with the rise in temperature, indicating that the sorption process of the Hg(II) ions onto **CMP** was more favourable at lower temperatures. In addition, the negative value of Δ*G*^0^ reveals that the sorption process was exothermic.

### 3.6. Possible mechanism of Hg(II) ion adsorption onto **CMP**

The adsorption mechanism of Hg(II) ions can be described as follows. Firstly, Hg(II) ions are transferred from the solution to the surface of **CMP** by the active functional groups from CS. Those active sites, such as hydroxyl (–OH) and amino (−NH_2_) groups, can form coordination bonds with metal ions via a chelation effect according to the following chelating mechanism [48–50]:

3.7 $$\begin{array}{rl} & \;(\text{CMP})-\text{N}{\text{H}}_{2}+\text{H}{\text{g}}^{2+}+(\text{CMP})-\text{N}{\text{H}}_{2}↔\text{(CMP) }-\text{N}{\text{H}}_{2}\cdots \text{H}{\text{g}}^{2+}\cdots {\text{H}}_{2}\text{N}-\text{(CMP) , } \end{array}$$

3.8 $$\begin{array}{rl} & \;(\text{CMP})-\text{OH}+\text{H}{\text{g}}^{2+}+(\text{CMP})-\text{OH}↔\text{(CMP) }-\text{OH}\cdots \text{H}{\text{g}}^{2+}\cdots \text{HO}-\text{(CMP) } \end{array}$$

3.9 $$\begin{array}{rl} \text{and}\; & \;(\text{CMP})-\text{N}{\text{H}}_{2}+\text{H}{\text{g}}^{2+}+(\text{CMP})-\text{OH}↔\text{(CMP) }-\text{N}{\text{H}}_{2}\cdots \text{H}{\text{g}}^{2+}\cdots \text{HO}-\text{(CMP) }\text{.} \end{array}$$

Secondly, when the surface active sites of **CMP** gradually reach the saturation state, Hg(II) ions may migrate from the **CMP** surface to the nano-pores by intraparticle diffusion and be adsorbed by the surface hydroxyl (−OH) groups and carboxylate anions (COO^−^) derived from PAA, forming Si–O–Hg–O–Si bridging species, –COO–Hg–OOC– bridging species or Si–O–Hg–OOC– bridging species according to the following mechanism [51,52]:

3.10 $$\begin{array}{rl} & \;(\text{CMP})-{\text{O}}^{-}+\text{H}{\text{g}}^{2+}+(\text{CMP})-{\text{O}}^{-}↔\text{(CMP) }-\text{O}-\text{Hg}-\text{O}-\text{(CMP), } \end{array}$$

3.11 $$\begin{array}{rl} & \;(\text{CMP})-\text{CO}{\text{O}}^{-}+\text{H}{\text{g}}^{2+}+(\text{CMP})-\text{CO}{\text{O}}^{-}↔\text{(CMP) }-\text{COO}-\text{Hg}-\text{OOC}-\text{(CMP) } \end{array}$$

3.12 $$\begin{array}{rl} \text{and}\; & \;(\text{CMP})-{\text{O}}^{-}+\text{H}{\text{g}}^{2+}+(\text{CMP})-\text{CO}{\text{O}}^{-}↔\text{(CMP) }-\text{O}-\text{Hg}-\text{OOC}-\text{(CMP) }\text{.} \end{array}$$

### 3.7. Comparison with other studies

Table 4 compares the best adsorption capacity of **CMP** with different common adsorbents for the removal of Hg(II) ions. It could be clearly seen that the adsorption capacity is higher than that of most adsorbents according to table 4. In addition, if we take into account the process characteristics of this work, for example, using cheap diatomite as the silica source can produce mesoporous materials, and, moreover, constructing organic–inorganic hybrid frameworks with polymer materials improves the properties of mesoporous materials, we can believe that **CMP** will be a good candidate for applications in heavy metal removal from wastewater.

**Table 4.** Comparison of the maximum adsorption capacity (*q*_max_) values of Hg(II) ions on **CMP** with those of other adsorbents reported in the literature.

adsorbent	*q*_max_(mg g^−1^)
polypyrrole/thiol-functionalized zeolite beta/MCM-41 type mesoporous silica nanocomposite	47 [51]
polyaniline/hexagonal mesoporous silica nanocomposite	51 [52]
mesoporous silica/polyacrylamide composite	177 [53]
CS/MCM-41-PAA nanocomposites (**CMP**)	164

## 4. Conclusion

In summary, the adsorbent (**CMP**) with a uniform adjustable pore structure, large specific surface area and rich in groups including –OH, –NH_2_ and –COOH was successfully designed and characterized. These characteristics could facilitate the contact of Hg(II) ions and active adsorption sites and attainment of rapid equilibrium adsorption. The results demonstrated that the maximum adsorption capacities reached 164 mg g^−1^ with a pH value of 4 and a contact time of 120 min at 298 K; which demonstrated excellent adsorption ability for Hg(II) ions. The adsorption behaviour followed pseudo-second-order kinetics, and the equilibrium data were well fitted by the Langmuir isotherm. The negative Δ*H*^0^ and Δ*G*^0^ suggested that the adsorption was a spontaneous exothermic process. These interesting findings might provide some new inspiration that using cheap diatomite as the silica source is a versatile approach for designing other mesoporous materials, and that constructing organic–inorganic hybrid frameworks with polymer materials enhances the adsorption properties of mesoporous materials.

## Data accessibility

This article does not contain any additional data.
